# Quercetin Protects against MPP^+^/MPTP-Induced Dopaminergic Neuron Death in Parkinson's Disease by Inhibiting Ferroptosis

**DOI:** 10.1155/2022/7769355

**Published:** 2022-09-05

**Authors:** Zhi-Hao Lin, Yi Liu, Nai-Jia Xue, Ran Zheng, Yi-Qun Yan, Zhong-Xuan Wang, Yao-Lin Li, Chang-Zhou Ying, Zhe Song, Jun Tian, Jia-Li Pu, Bao-Rong Zhang

**Affiliations:** Department of Neurology, Second Affiliated Hospital, School of Medicine, Zhejiang University, Hangzhou, Zhejiang 310009, China

## Abstract

Ferroptosis, a novel form of regulated cell death, is caused by accumulation of lipid peroxides and excessive iron deposition. This process has been linked to the death of dopaminergic neurons in substantia nigra compacta (SNc) of Parkinson's disease (PD) patients. Quercetin (QCT), a natural flavonoid, has multiple pharmacological activities. However, it has not been established whether QCT can protect against dopaminergic neuron death by inhibiting ferroptosis. In this study, we investigated the potential antiferroptotic effects of QCT in cellular models established using specific ferroptosis inducers (Erastin and RSL-3) and MPP^+^. The effects were also explored using MPTP-induced PD mouse models. The cell counting kit-8 (CCK-8) assay was performed to assess cell viability. Variations in mitochondrial morphology were evaluated by transmission electron microscopy (TEM) while the mitochondrial membrane potential, mass, and ROS were measured by fluorescent probes. Lipid peroxidation levels were assayed through measurement of lipid ROS, MDA, GSH, and SOD levels. The effects of QCT on MPTP-induced behavioral disorders were examined by rotarod and open field tests. *In vitro* and *in vivo*, QCT significantly inhibited ferroptosis by activating the nuclear factor erythroid 2-related factor 2 (Nrf2) protein. Additionally, QCT ameliorated motor behavioral impairments and protected against the loss of dopaminergic neurons in MPTP-induced PD models. Interestingly, Nrf2 knockdown alleviated the protective effects of QCT against ferroptosis. In conclusion, these results demonstrate that ferroptosis is involved in MPP^+^/MPTP-induced PD, and QCT inhibits ferroptosis by activating the Nrf2 protein. Therefore, QCT is a potential agent for preventing the loss of dopaminergic neurons by targeting ferroptosis.

## 1. Introduction

Globally, Parkinson's disease (PD) is one of the most common neurodegenerative diseases, affecting about 2% of individuals aged over 60 years. It is characterized by a wide spectrum of motor and nonmotor symptoms, including resting tremors, bradykinesia, rigidity, cognitive impairments, and sleep disorders [1]. Evidence shows that PD is caused by genetic and environmental factors [2]; however, its pathogenesis has not been conclusively determined. This has posed a huge challenge to the clinical treatment of this disorder. Thus, there is an urgent need to identify effective agents for the management of PD. The major neuropathological hallmark of PD is dopaminergic neuronal loss in the substantia nigra compacta (SNc) [3]. Apoptosis has an important role in dopaminergic neuronal death [4]. However, the predominant type of the death of nigrostriatal dopaminergic neurons has not been conclusively determined.

Ferroptosis is a newly discovered form of programmed cell death that differs from apoptosis, pyroptosis, and necroptosis. This type of cell death was first described by Dixon et al. in 2012. It is characterized by iron-dependent lipid peroxidation [5, 6] and is involved in various disorders including cancer, neurodegenerative diseases, stroke, and acute kidney injury [7]. In ferroptotic cells, the ultrastructure of mitochondria is altered. Specifically, mitochondria appear smaller than normal and showed increased mitochondrial membrane density, decreased mitochondrial crista, and rupture of the outer membrane [5, 8]. Due to excessive iron deposition, increased lipid peroxidation, and decreased glutathione levels in the SNc of PD patients, it is likely that ferroptosis is associated with and contributes to PD pathogenesis [9]. In a previous study, ferroptosis-specific inhibitor, ferrostatin-1 (Fer-1), protected dopaminergic neurons and alleviated behavioral impairments in MPTP PD models [10]. Other PD-related *in vivo* and *in vitro* models (SNCA^A53T^ mice, 6-OHDA, MPP^+^, and paraquat) have shown the involvement of ferroptosis in PD [11–14]. Therefore, inhibition of ferroptosis may be a promising strategy for PD treatment.

Natural products can be used to develop novel agents for PD treatment due to their multiple bioactive properties [15, 16]. Quercetin (QCT), a flavonoid compound, ubiquitously exists in various vegetables and fruits and in traditional Chinese herbal medicines [17]. Pharmacologically, QCT exhibits anti-inflammatory, antioxidant, anticancer, antiapoptotic, and antiferroptotic functions in various diseases [17–19]. Moreover, QCT can improve mitochondrial dysfunction, which is a feature of ferroptosis [5, 20, 21]. Mechanistically, QCT exerts its neuroprotective effects by inhibiting microglial activation [22]. However, it has not been established whether QCT protects against the death of dopaminergic neurons by inhibiting ferroptosis.

Nuclear factor erythroid 2-related factor 2 (Nrf2), an essential transcription factor, has anti-inflammatory and antioxidant functions. Moreover, it has therapeutic effects in many neurodegenerative diseases, including PD [23]. Interestingly, Nrf2 acts as a transcription factor that regulates the expression of ferroptosis-related proteins, such as glutathione peroxidase 4 (GPX4), solute carrier family 7 member 11 (SLC7A11), and ferritin heavy chain (FTH) thereby inhibiting the occurrence of ferroptosis [24]. Activation of Nrf2 protects against mitochondrial damage and prevents ROS generation, which are vital factors associated with ferroptosis induction [24, 25]. These results show that Nrf2 modulates PD progression by regulating ferroptosis.

In this study, we investigated whether QCT inhibits ferroptosis in PD by activating the Nrf2 protein. Our findings showed that QCT suppresses cell death in PD by activating the Nrf2 protein leading to inhibition of ferroptosis. Moreover, QCT alleviated *in vivo* MPTP-induced behavioral impairment and dopaminergic neuronal death in SNc by inhibiting ferroptosis.

## 2. Materials and Methods

### 2.1. Chemicals and Reagents

Quercetin (QCT, Q4951) for *in vivo* assays and 1-methyl-4-phenylpyridinium (MPP^+^, D048) and MPTP-HCL (M0896) were obtained from Sigma-Aldrich (St. Louis, MO, USA). Quercetin (HY-18085) for *in vitro* assays and Rhodamine 123 (HY-D0816) were obtained from MedChemExpress (MCE, Monmouth Junction, NJ, USA). Erastin (S7242), ferrostatin-1 (Fer-1, S7243), and liproxstatin-1 (Lip-1, S7699) were purchased from Selleck.cn (Houston, TX, USA). BODIPY 581/591 C11 probe (D3861) was purchased from Invitrogen (Rockford, USA). MitoSOX Red (MT14), Malondialdehyde (MDA) Assay Kit (M496), and GSH Assay Kit (G263) were obtained from Dojindo (Kumamoto, Japan). MitoTracker Green (C1048), ATP Assay Kit (S0026), and SOD Assay Kit (S0101) were purchased from Beyotime Biotechnology (Jiangsu, China). Cell counting kit-8 (CCK-8, K1018) was purchased from APExBIO (Houston, TX, USA).

For western blotting experiments, the antibodies used were as follows: anti-GPX4 Ab (1 : 1000, Abcam, ab125066), anti-SLC7A11 Ab (1 : 1000, Abcam, ab175186), anti-Nrf2 Ab for humans (1 : 1000, Abcam, ab62352), anti-Nrf2 Ab for mouse and rat (1 : 1000, ABclonal, A11159), anti-FTH Ab (1 : 1000, Affinity, DF6278), anti-TH Ab (1 : 1000, Cell Signaling Technology, 58844), anti-GAPDH Ab (1 : 3000, HUABIO, EM1101), anti-Lamin B1 ab (1 : 1000, HUABIO, ET1606-27), horseradish peroxidase- (HRP-) conjugated goat anti-rabbit Ab (1 : 5000, HUABIO, HA1001), and HRP conjugated goat anti-mouse Ab (1 : 5000, HUABIO, HA1006). For immunofluorescent experiments, the antibodies used were as follows: anti-Nrf2 Ab for human (1 : 100, Abcam, ab62352), anti-Nrf2 Ab for mouse (1 : 100, ABclonal, A11159), anti-TH Ab (1 : 500, Cell Signaling Technology, 58844); and Alexa fluor-488 conjugated anti-rabbit (1 : 500, Invitrogen, A-21206).

### 2.2. Cell Cultures and Treatments

The human neuroblastoma M17 cell line was provided by Dr. Shengdi Chen at Ruijin Hospital of Shanghai Jiao Tong University (Shanghai, China). Cells were cultured in Opti-MEM supplemented with 10% FBS and 1% penicillin/streptomycin and incubated in a 5% CO_2_ atmosphere at 37°C. The M17 cells were treated with or without QCT (10 *μ*M), Fer-1 (1 *μ*M), or Lip-1 (200 nM) for 1 h, followed by Erastin (1 *μ*M) treatment for 12 or 24 h.

Rat pheochromocytoma PC12 cells were obtained from the Cell Bank of the Chinese Academy of Sciences (Beijing, China). Cells were cultured in RPMI 1640 medium supplemented with 10% FBS and 1% penicillin/streptomycin and incubated in a 5% CO_2_ atmosphere at 37°C. The PC12 cells were treated with or without QCT (10 *μ*M) and Fer-1 (1 *μ*M) for 1 h, followed by MPP^+^ (0.5 mM) treatment for 24 h.

The human neuroblastoma SH-SY5Y cell line was obtained from the Cell Bank of the Chinese Academy of Sciences (Beijing, China). Cells were cultured in DMEM/F12 medium supplemented with 10% FBS and 1% penicillin/streptomycin and incubated in a 5% CO_2_ atmosphere at 37°C. The SH-SY5Y cells were treated with or without QCT (10 *μ*M), Fer-1 (1 *μ*M), or Lip (200 nM) for 1 h, followed by Erastin (1 *μ*M) treatment for 24 h.

### 2.3. Cell Viability Assay

The viability of M17 and PC12 cells was assessed by cell counting kit-8 (CCK-8) (APExBIO, Houston, USA) following instructions provided by the manufacturer. Briefly, cells were seeded in 96-well plates and cultured overnight. After treatment, 100 *μ*L medium containing 10 *μ*L CCK-8 solution was added to each well and incubated for 2-4 h. Absorbance was measured by a microplate reader (Molecular Devices SpectraMax 190, USA) at 450 nm.

### 2.4. Transmission Electron Microscopy (TEM)

M17 and PC12 cells were fixed in 2.5% glutaraldehyde at 4°C overnight. Subsequently, the cells were refixed in 1% osmium tetroxide for 1 h, stained with 2% uranyl acetate for 30 min, and dehydrated in a series of graded ethanol. Next, the samples were embedded in 100% acetone for 2 h, sliced into 90 nm ultrathin sections, and stained with 4% uranyl acetate and 1% lead citrate. The TEM (Tecnai G2 Spirit 120 kV, Netherlands) was used to evaluate the ultrastructure of cells.

### 2.5. Lipid ROS Assay

Intracellular lipid ROS levels were measured using the BODIPY 581/591 C11 reagent (Invitrogen) following the manufacturer's instructions. The M17 cells were seeded in six-well plates and treated with Erastin, with or without QCT or Fer-1 for 12 h. They were then washed with PBS twice and incubated with 5 *μ*M BODIPY 581/591 C11 reagent for 30 min in an incubator controlled to a temperature of 37°C. Cells were harvested and washed twice with PBS and then resuspended in 500 *μ*L of PBS. They were strained through a 70 *μ*m strainer and examined using a flow cytometer (Beckman CytoFLEX) equipped with a 488 nm laser. Data were analyzed using the FlowJo software.

### 2.6. Immunofluorescent and Confocal Microscopy

The M17 and PC12 cells were seeded in 24-well glass slides. After treatment, they were fixed in 4% paraformaldehyde (PFA) for 30 min at room temperature, washed thrice using PBS, permeabilized using 0.1% Triton X-100, blocked using 5% BSA for 1 h, and incubated overnight with primary antibodies at 4°C. The cells were then incubated with Alexa fluor-488 conjugated anti-rabbit antibody (1 : 500) for 1 h at room temperature, and the nuclei were stained with DAPI (Beyotime Biotechnology). Samples were imaged through TSC SP8 confocal microscopy (Leica).

### 2.7. Mitochondrial Functions

Mitochondrial membrane potential was measured using Rhodamine 123. PC12 cells were seeded in six-well plates. After treatment, the cells were incubated for 30 min at 37°C with 10 *μ*M Rhodamine 123 (MCE) and washed thrice using PBS.

Mitochondrial mass was measured using MitoTracker Green (Beyotime Biotechnology). Briefly, PC12 cells were seeded in six-well plates, treated, incubated for 30 min at 37°C with 200 nM MitoTracker Green, and washed thrice using PBS.

Mitochondrial ROS was measured using MitoSOX Red. Briefly, PC12 cells were seeded in six-well plates. After treatment, the cells were incubated for 30 min at 37°C with 10 *μ*M MitoSOX Red (Dojindo) and washed thrice using PBS. Results of these three experiments were analyzed using a flow cytometer (Beckman CytoFLEX) and analyzed using FlowJo software.

Intracellular ATP levels were measured using the ATP Assay Kit (Beyotime Biotechnology). Briefly, PC12 cells were seeded in six-well plates. After treatment, the cells were lysed using a lysis buffer. The harvested cells were centrifuged at 12,000 × g for 5 min at 4°C. Next, 20 *μ*L of the supernatant or standards was added to 100 *μ*L of the ATP testing solution in a light-proof 96-well plate, Finally, the concentration of ATP was measured by a multimode plate reader.

### 2.8. siRNA Transfections

Nrf2 gene knockdown was achieved using siRNA. PC12 cells were seeded in 6 or 96-well plates and incubated overnight. Lipofectamine 3000 (Invitrogen) was used for transient siRNA transfection following the manufacturer's instructions. Briefly, 5 *μ*L of 20 *μ*M siRNA and 125 *μ*L Opti-MEM were placed in a tube while 5 *μ*L Lipofectamine 3000 and 125 *μ*L Opti-MEM were placed in another tube. The two tubes were gently mixed and incubated for 15 min at room temperature. Next, the mixture was transferred into a 6-well plate. The siRNA was purchased from GenePharma (Shanghai, China), and it had the following target sequence: GGAGAGGGAAGAAUAAAGUTT (sense); ACUUUAUUCUUCCCUCUCCTT (antisense).

### 2.9. Nuclear Protein Extraction

The M17 and PC12 cells were seeded in six-well plates and treated. The Nuclear and Cytoplasmic Protein Extraction Kit (Beyotime Biotechnology) was used for nuclear protein extraction as instructed by the manufacturer.

### 2.10. Western Blot Assay

Cells and brain tissues were lysed using the RIPA lysis buffer (Beyotime Biotechnology) containing 1% protease inhibitor cocktail (Invitrogen) for 30 min and centrifuged at 12000 × g, 4°C for 20 min. Protein concentration in the samples was determined using the BCA Assay Kit (Invitrogen). The samples were mixed with a loading buffer and denatured by heating at 100°C for 5 min. Next, 20 *μ*g of total and nuclear protein was separated using 8-12% SDS-PAGE and transferred to polyvinylidene difluoride membranes (Merck Millipore), which were blocked with 5% skim milk in 0.1% Tween-20/Tris-buffered saline for 1 h at room temperature. Membranes were incubated overnight in the presence of primary antibodies at 4°C. They were subsequently incubated with HRP-conjugated secondary antibodies (HUABIO) for 1 h at room temperature. Finally, immunoblots were detected by a chemiluminescence reagent (Invitrogen) and analyzed using ImageJ.

### 2.11. *In Vivo* Assays

Male C57BL/6 mice (8-10 weeks) were purchased from SLAC Laboratory Animal Co., Ltd (Shanghai, China) and housed in a temperature- and humidity-controlled environment under a 12-h light/dark cycle with free drinking water and food. After 1 week of acclimation, mice were randomized into three groups: control (Ctrl) group, MPTP group, and MPTP QCT group (mice were intraperitoneally injected 60 mg/kg/day QCT 3 days before MPTP treatment and over the 5 MPTP injection days) [22]. Mice were intraperitoneally injected with 30 mg/kg/day MPTP (dissolved in 0.9% saline) for 5 consecutive days to establish subacute PD mouse models [26]. Control mice were intraperitoneally injected with an equal volume of 0.9% saline. The QCT was dissolved in dimethyl sulfoxide (DMSO) and diluted in 0.9% normal saline to obtain a final concentration of 5 mg/mL solution containing 5% DMSO. Mice received QCT (60 mg/kg/day, i.p.) treatment 3 days prior to treatment with MPTP and over the 5 MPTP injection days in PD model. Animal procedures were performed in accordance with the Guide for the Care and Use of Laboratory Animals as published by the National Institutes of Health. The Institutional Ethics Committee of the Second Affiliated Hospital, Zhejiang University School of Medicine, approved this study.

### 2.12. Behavioral Tests

For the rotarod test, mice were placed on an accelerating rotating rod with the speed increased from 4 to 40 r.p.m. within 300 s. Prior to the test, mice were trained for 3 days. All mice were tested thrice, separated by 20 min intertrial intervals. Latency to fall was recorded. Data are presented as the mean value of three trials.

For the open field test, mice were transferred to the testing room 1 h before testing for adaptation. Next, mice were placed in the middle of the cubic box under evenly distributed illumination for 5 min. The total movement distance for the 5 min period was recorded using a SMART video tracking software (Smart 3.0).

### 2.13. Immunofluorescence of Brain Slices

Mice were deeply anaesthetized with 1% sodium pentobarbital (0.1 mg/g, i.p.), and perfused with PBS, followed by 4% PFA. Next, whole brains were rapidly removed and placed into a 4% PFA solution at 4°C for 24-48 h after which they were placed in a sucrose solution (30%) for complete dehydration for at least 48 h. After the brains had sunk to the bottom, they were embedded in an OCT compound and coronally sliced into 30 *μ*m serial sections using a cryostat microtome (Leica CM1950). For immunofluorescence staining, brain slices were washed three times using PBS, blocked, and permeabilized using a QuickBlot™ Blocking Buffer (Beyotime Biotechnology) for 30 min at room temperature. The brain slices were then incubated overnight with a primary antibody at 4°C, washed thrice using PBS, and incubated for 1 h with corresponding fluorescent secondary antibodies at room temperature. It was subsequently washed thrice and stained with DAPI (Beyotime Biotechnology). Finally, images were acquired using a fluorescent microscope (Leica DM6B).

### 2.14. Measurement of Lipid Peroxidation Markers

Malondialdehyde (MDA), glutathione (GSH), and superoxide dismutase (SOD) levels were measured using commercial kits (MDA: Dojindo, M496; GSH: Dojindo, C1048; SOD, Beyotime Biotechnology, S0101). Briefly, the SN tissue was homogenized at low temperatures using the lysis solution in the kits. Finally, the BCA Assay Kit (Invitrogen) was used to determine protein concentration.

### 2.15. Statistical Analysis

Data are expressed as mean ± SEM. Comparison of means between and among groups was performed using the Student *t*-test or one-way ANOVA followed by Tukey's post hoc test, respectively. All statistical analyses were conducted using Prism 7.0 software (San Diego, CA, USA). *p* ≤ 0.05 was set as the threshold for significance.

## 3. Results

### 3.1. QCT Inhibited Erastin- and RSL3-Induced Ferroptosis

**Figure 1:**
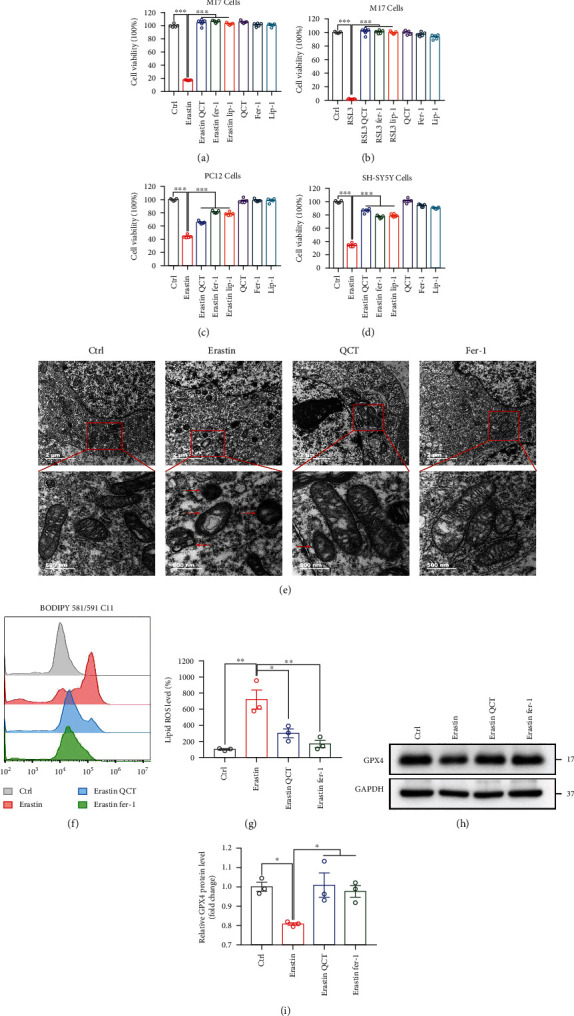
QCT significantly inhibited Erastin- and RSL3-induced ferroptosis in M17, PC12, and SH-SY5Y cells. (a–d) Viabilities of M17, PC12, and SH-SY5Y cells after treatment with QCT (10 *μ*M), Fer-1 (1 *μ*M), and Lip-1 (200 nM) for 1 h, respectively, followed by Erastin (1 *μ*M, 24 h) or RSL3 (1 *μ*M, 12 h) treatments (*n* = 5). (e) Representative transmission electron microscope images for M17 cells treated with or without QCT (10 *μ*M) or Fer-1 (1 *μ*M, 1 h), followed by Erastin (2 *μ*M, 12 h) treatment. Red arrows indicate shrunken mitochondria. Scale bars as indicated. (f, g) The M17 cells were treated with QCT (10 *μ*M) or Fer-1 (1 *μ*M) for 1 h, followed by Erastin (2 *μ*M) for 24 h, and the lipid ROS assayed by flow cytometry using C11-BODIPY (*n* = 3). (h) Representative immunoblots of the GPX4 protein from M17 cells treated with or without QCT (10 *μ*M, 1 h), or Fer-1 (1 *μ*M, h), followed by Erastin (1 *μ*M, 12 h) treatment. (i) Quantification of GPX4 protein levels (*n* = 3). Data are presented as mean ± SEM. One-way ANOVA followed by Tukey's post hoc test. ^∗^*p* < 0.05, ^∗∗^*p* < 0.01, and ^∗∗∗^*p* < 0.001.

The CCK-8 assay was performed to determine optimal experimental concentration of QCT. High QCT concentration caused cytotoxic effects, which was consistent with findings from a previous report [19]. Therefore, we chose 10 *μ*M QCT as the ideal concentration in all experiments (Figs. S1A-C). Erastin and RSL-3, which directly inhibit the cystine-glutamate antiporter system and GPX4, respectively, are inducers of ferroptosis [27, 28]. In this study, we established that RSL-3 or Erastin caused significant death rate of M17 cells which was completely reversed by QCT, Fer-1, or Lip-1 treatments (Figures 1(a) and 1(b)). Similarly, Erastin-treated PC12 and SH-SY5Y cells were rescued by QCT, Fer-1, or Lip-1 treatments (Figures 1(c) and 1(d)). Then, we evaluated whether QCT could reverse morphological changes in ferroptotic cells. Representative transmission electron microscopy (TEM) images showed shrunken mitochondria with increased membrane densities in Erastin-treated M17 cells (as indicated by the red arrow). However, these effects were alleviated with QCT or Fer-1 treatments (Figure 1(e)). Given that lipid ROS is a key ferroptosis characteristic, we assessed lipid ROS generation using a BODIPY 581/591 C11 probe. We found that QCT suppressed the overproduction of lipid ROS, similar to Fer-1 (Figures 1(f) and 1(g)). Moreover, Erastin treatment downregulated GPX4 protein levels. However, GPX4 protein levels were upregulated following treatment with QCT or Fer-1 (Figures 1(h) and 1(i)). These findings support the hypothesis that QCT protects M17, PC12, and SH-SY5Y cells against Erastin-induced ferroptosis.

### 3.2. QCT Inhibited Erastin-Induced Ferroptosis in M17 Cells by Activating the Nrf2 Protein

**Figure 2:**
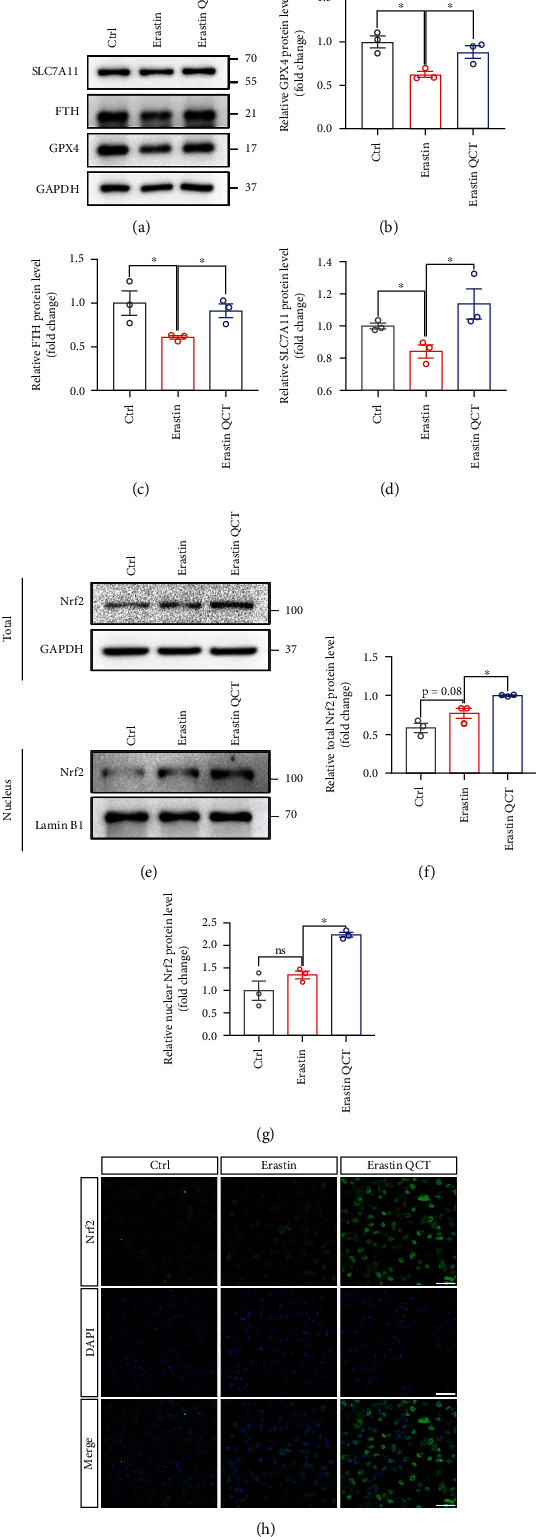
QCT inhibited Erastin-induced ferroptosis in M17 cells by activating the Nrf2 pathway. (a) Representative immunoblots for SLC7A11, FTH, and GPX4 proteins from M17 cells treated with or without QCT (10 *μ*M, 1 h), followed by Erastin (1 *μ*M, 12 h) treatment. (b–d) Quantification of SLC7A11, FTH, and GPX4 protein levels (*n* = 3). (e, f) Representative immunoblots for nuclear and total Nrf2 protein levels from M17 cells treated with or without QCT (10 *μ*M, 1 h), followed by Erastin (1 *μ*M, 12 h) treatment. (f, g) Quantification of nuclear and total Nrf2 protein levels (*n* = 3). Immunofluorescence analysis of M17 cells treated with or without QCT (10 *μ*M, 1 h), followed by Erastin (1 *μ*M, 12 h) treatment, stained with Nrf2 antibody (green) and DAPI (blue). Scale bar = 50 *μ*m. Data are presented as mean ± SEM. One-way ANOVA followed by Tukey's post hoc test. ^∗^*p* < 0.05, ^∗∗^*p* < 0.01, and ^∗∗∗^*p* < 0.001; ns: no significant difference.

It has been reported that QCT activates the Nrf2 pathway to counteract oxidation and inflammation [29, 30]. Moreover, as a transcriptional factor, Nrf2 regulates ferroptosis-related protein levels. Thus, we hypothesized that QCT activates Nrf2 proteins to inhibit Erastin-induced ferroptosis. Nuclear protein levels from whole-cell lysates were extracted after which the expression of total and nuclear Nrf2 protein was assessed by western blotting. After simultaneous treatments with QCT, total and nuclear Nrf2 proteins were upregulated, compared to Erastin treatment alone. Differences in total and nuclear Nrf2 protein levels between the Erastin and control group were insignificant (Figures 2(e)–2(g)). Representative immunofluorescence images showed that QCT promoted Nrf2 protein expression and its translocation into the nucleus (Figure 2(h)). Next, we investigated the changes in expression of ferroptosis-related proteins downstream of the Nrf2 protein. After Erastin treatment, protein levels of GPX4, FTH, and SLC7A11 were downregulated in M17 cells, while QCT significantly inhibited these changes (Figures 2(a)–2(d)). In summary, QCT inhibited Erastin-induced ferroptosis in M17 cells.

### 3.3. QCT Protected against MPP^+^-Induced Cell Death by Inhibiting Ferroptosis

**Figure 3:**
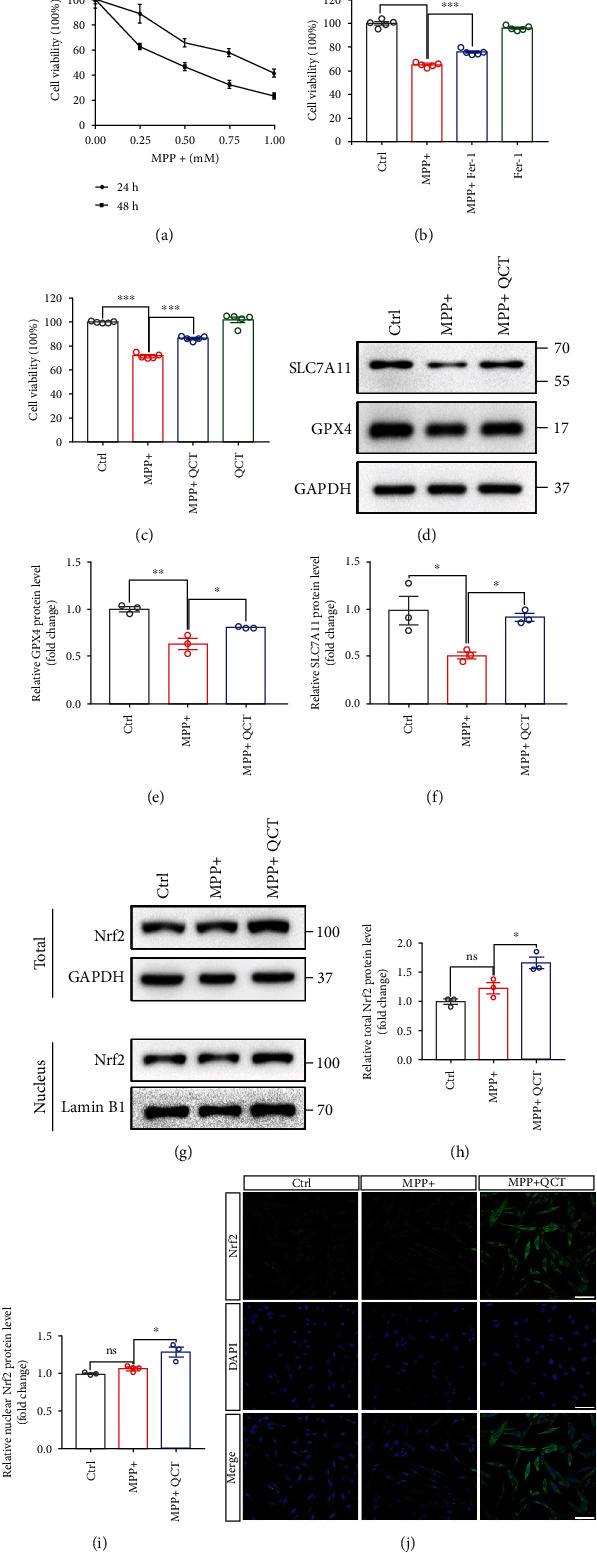
QCT inhibited MPP^+^-induced ferroptosis in PC12 cells by activating the Nrf2 pathway. (a) Viabilities of PC12 cells treated with 0.25, 0.5, 0.75, and 1 mM MPP^+^ for 24 h or 48 h (*n* = 5). (b) Viability of PC12 cells treated with or without Fer-1 (1 *μ*M, 1 h), followed by MPP^+^ (0.5 mM, 24 h) treatment (*n* = 5). (c) Viability of PC12 cells treated with or without QCT (10 *μ*M, 1 h), followed by MPP^+^ (0.5 mM, 24 h) treatment (*n* = 5). (d) Representative immunoblots for SLC7A11 and GPX4 proteins from PC12 cells treated with or without QCT (10 *μ*M, 1 h), followed by MPP^+^ treatment (0.5 mM, 24 h). (e, f) Quantification of SLC7A11 and GPX4 protein levels (*n* = 3). (g) Representative immunoblots for nuclear and total Nrf2 proteins from PC12 cells treated with or without QCT (10 *μ*M, 1 h), followed by MPP^+^ (0.5 mM, 24 h) treatment. (h, i) Quantification of nuclear and total Nrf2 protein levels (*n* = 3). (j) Immunofluorescent analysis from PC12 cells treated with or without QCT (10 *μ*M, 1 h), followed by MPP^+^ (0.5 mM, 24 h) treatment. Scale bar = 50 *μ*m. Data are presented as mean ± SEM. One-way ANOVA followed by Tukey's post hoc test. ^∗^*p* < 0.05, ^∗∗^*p* < 0.01, and ^∗∗∗^*p* < 0.001; ns: no significant difference.

First, PC12 cells were treated with MPP^+^ at various doses and times to establish *in vitro* PD cell models [13, 31]. Increasing MPP^+^ doses and exposure time decreased the survival of PC12 cells (Figure 3(a)). Then, PC12 cells were treated with or without Fer-1 (1 *μ*M, 1 h), followed by MPP^+^ (0.5 mM, 24 h) treatment. Fer-1 treatment rescued the MPP^+^-induced cell death (Figure 3(b)). These results were in tandem with previous studies [13, 31], which indicated that MPP^+^ induced ferroptosis in PC12 cells. To validate whether QCT could rescue MPP^+^-induced ferroptosis, PC12 cells were treated with or without QCT, followed by MPP^+^ treatment. It was established that QCT inhibited MPP^+^-induced cell death (Figure 3(c)). Moreover, GPX4 and SLC7A11 protein levels in PC12 cells were downregulated by MPP^+^ treatment, similar to previous studies [31, 32]. However, QCT treatment significantly inhibited these changes (Figures 3(d)–3(f)). Total and nuclear Nrf2 protein levels were upregulated by QCT and MPP^+^ treatments, compared to MPP^+^ treatment alone (Figures 3(g)–3(i)). There were no marked differences in total and nuclear Nrf2 protein levels between the MPP^+^ and control groups. Immunofluorescence analysis showed that QCT upregulated Nrf2 levels and its translocation into the nucleus (Figure 3(j)). These findings showed that QCT inhibited MPP^+^-induced ferroptosis.

### 3.4. QCT Protected against MPP^+^-Induced Mitochondrial Dysfunction in PC12 Cells

**Figure 4:**
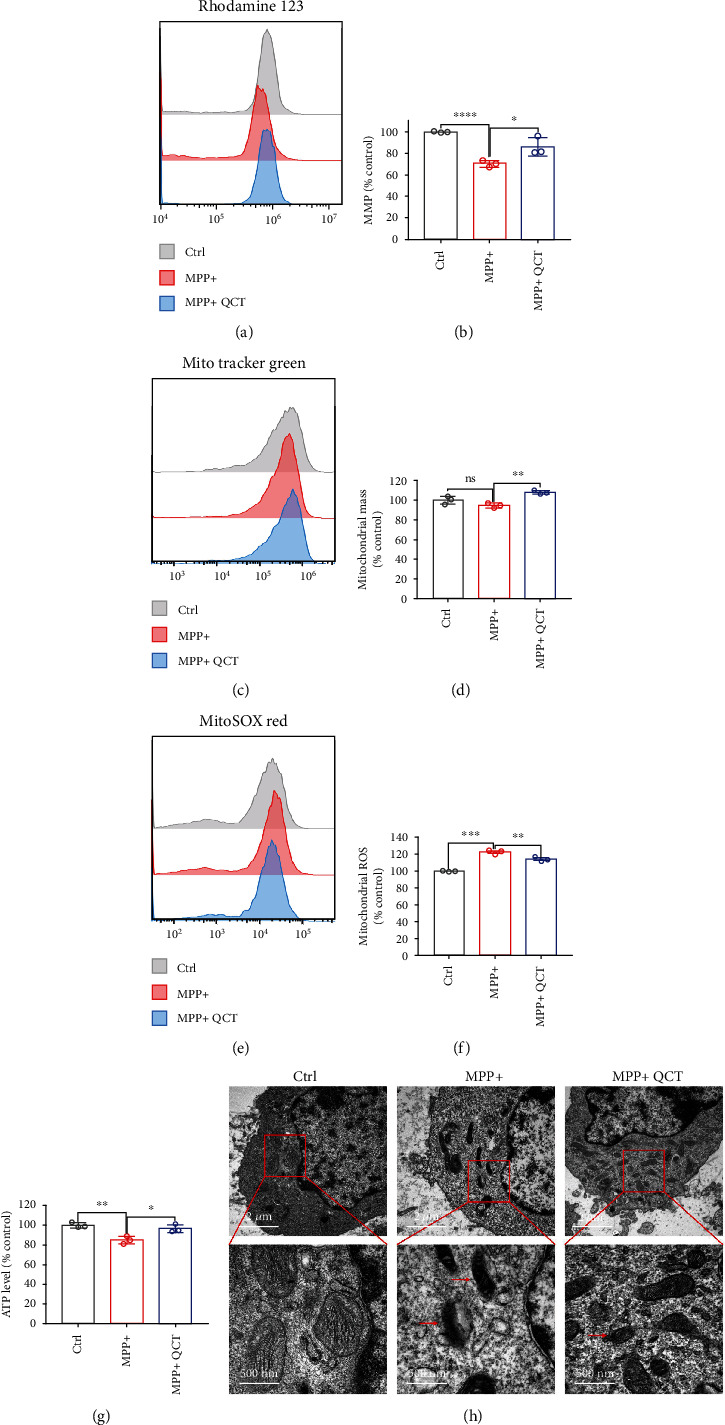
QCT alleviated mitochondrial dysfunction in MPP^+^-treated PC12 cells. (a, b) Mitochondrial membrane potential was assayed by flow cytometry using Rhodamine 123 (*n* = 3). (c, d) Mitochondrial mass was assayed by flow cytometry using MitoTracker Green (*n* = 3). (e, f) Mitochondrial ROS was assayed by flow cytometry using MitoSOX Red (*n* = 3). (G) Intracellular ATP levels in PC12 cells treated with or without QCT (10 *μ*M, 1 h), followed by MPP^+^ (0.5 mM, 24 h) treatment, were assayed by the ATP Assay Kit (*n* = 3). (h) Representative transmission electron microscopy images for PC12 cells treated with or without QCT (10 *μ*M, 1 h), followed by MPP^+^(0.5 mM, 24 h) treatment. Red arrows indicate shrunken mitochondria. Scale bars as indicated. Data are presented as the mean ± SEM. One-way ANOVA followed by Tukey's post hoc test. ^∗^*p* < 0.05, ^∗∗^*p* < 0.01, and ^∗∗∗^*p* < 0.001.

The mitochondria are major ROS sources and are closely associated with ferroptosis [21, 33]. Thus, we evaluated the changes in mitochondrial functions in response to MPP^+^ treatment with or without QCT treatment. The mitochondrial membrane potential was downregulated by MPP^+^ treatment; however, it was inhibited by QCT treatment (Figures 4(a) and 4(b)). As shown in Figures 4(c) and 4(d), mitochondrial mass was slightly reduced by MPP^+^ treatment, which was improved by QCT treatment. Mitochondrial ROS generation was markedly increased by MPP^+^ treatment; however, QCT treatment suppressed RPS levels (Figures 4(e) and 4(f)). Subsequently, we evaluated the effects of MPP^+^ and QCT on intracellular ATP levels. As shown in Figure 4(g), QCT treatment rescued the MPP^+^-mediated decrease in intracellular ATP levels. To investigate mitochondrial changes, TEM was performed to observe changes in mitochondrial ultrastructure in PC12 cells. The TEM images showed smaller than normal mitochondria, reduced or absent mitochondrial cristae, or ruptured outer membranes after MPP^+^ treatment (as indicated by the red arrow). However, QCT markedly improved these changes in the mitochondria (Figure 4(h)). Therefore, QCT improved mitochondrial functions following MPP^+^ treatment.

### 3.5. Nrf2 Knockdown Compromised the Protective Role of QCT in Ferroptosis

**Figure 5:**
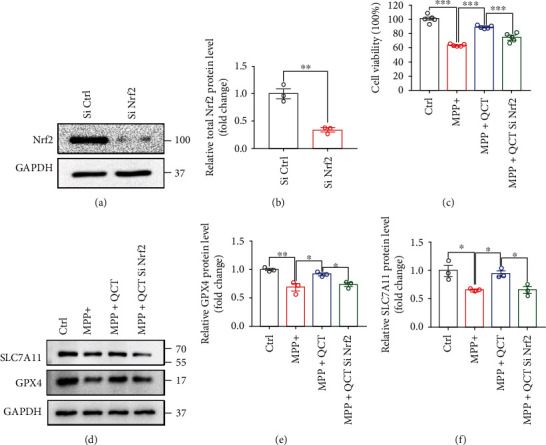
Nrf2 knockdown compromised the protective effects of QCT. (a) Representative immunoblots for PC12 cells transfected with Ctrl siRNA or Nrf2 siRNA. (b) Quantification of Nrf2 protein levels (*n* = 3). (c) Cell viability was assayed for transfected PC12 cells subjected to QCT (10 *μ*M, 1 h), before being treated with MPP^+^ (0.5 mM, 24 h) (*n* = 5). (d) Representative immunoblots for SLC7A11 and GPX4 proteins from PC12 cells transfected with Nrf2 siRNA for 48 h and then treated with QCT (10 *μ*M, 1 h) before being subjected to MPP^+^ (0.5 mM, 24 h). (e, f) Quantification of SLC7A11 and GPX4 protein levels (*n* = 3). Data are presented as the mean ± SEM. Student's *t*-test and one-way ANOVA followed by Tukey's post hoc test. ^∗^*p* < 0.05, ^∗∗^*p* < 0.01, and ^∗∗∗^*p* < 0.001.

To determine whether QCT relies on the Nrf2 pathway to inhibit ferroptosis, we knocked down Nrf2 protein expression and used control siNrf2 and Nrf2 siNrf2 to determine knockdown effects. As shown in Figures 5(a) and 5(b), Nrf2 siRNA significantly suppressed Nrf2 protein expression following transfection. In further experiments, we investigated the effects of Nrf2 knockdown on the efficacy of QCT. Nrf2 knockdown reduced the therapeutic effects of QCT, as revealed by the CCK-8 assay (Figure 5(c)). Biologically, GPX4 and SLC7A11 proteins are important markers of ferroptosis. Thus, we determined the changes in expression of these proteins after Nrf2 siRNA transfection. Protein expression of GPX4 and SLC7A11 was downregulated following Nrf2 siRNA transfection when compared to control siRNA transfection under the same MPP^+^ and QCT treatment (Figures 5(d)–5(f)). These results imply that QCT activated the Nrf2 protein to exert its antiferroptotic effects.

### 3.6. QCT Attenuated Behavioral Disorders and Protected the Dopaminergic Neurons in the MPTP Models

**Figure 6:**
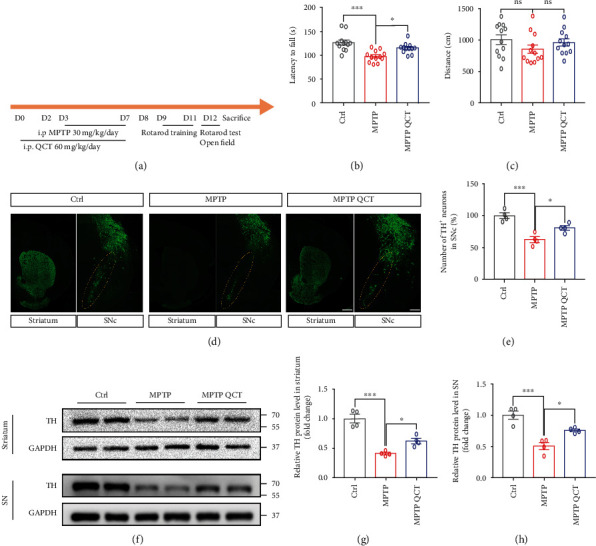
QCT attenuated behavioral disorders and protected the dopaminergic neurons in PD mouse models. (a) Diagram of the experimental procedure. Mice were intraperitoneally (i.p.) injected with MPTP (30 mg/kg/day) or saline for 5 consecutive days. Three days before treatment with MPTP, mice received QCT (60 mg/kg/day, i.p.) treatment. All animals were trained for 3 days and tested on day 12. (b) Rotarod tests were conducted for Ctrl, MPTP, and MPTP QCT groups (*n* = 12). (c) Open field test was conducted for Ctrl, MPTP, and MPTP QCT groups (*n* = 12). (d) Representative immunofluorescent staining of tyrosine hydroxylase (TH) in the substantia nigra compacta (SNc) and striatum. Scale bar = 200 *μ*m. (e) Quantification of relative TH-neurons in SNc (*n* = 4). (f) Representative immunoblots of the TH protein in SN and striatum. (g, h) Quantification of TH protein levels in SNc and striatum (*n* = 4). Data are presented as the mean ± SEM. One-way ANOVA followed by Tukey's post hoc test. ^∗^*p* < 0.05, ^∗∗^*p* < 0.01, and ^∗∗∗^*p* < 0.001; ns: no significant difference.

To elucidate on *in vivo* QCT effects, behavioral tests were performed (Figure 6(a)). In the rotarod test, QCT significantly relieved the decreased latency to fall after MPTP treatment (Figure 6(b)). In the open field test, there were no marked differences among the three groups (Figure 6(c)). To assess the neuroprotective effects of QCT, western blotting and tyrosine hydroxylase (TH) immunofluorescence were used to explore the loss of dopaminergic neurons in the SNc and striatum. As shown in Figures 6(d) and 6(e), following MPTP injection, QCT partially alleviated the loss of TH-positive neurons in the SNc and striatum. Moreover, TH protein levels were downregulated by MPTP injection in the SNc and striatum; however, QCT treatment markedly inhibited this change (Figures 6(f)–6(h)). Collectively, these findings indicate that QCT protected dopaminergic neurons in the MPTP model.

### 3.7. QCT Inhibited *In Vivo* Ferroptosis by Activating the Nrf2 Protein

**Figure 7:**
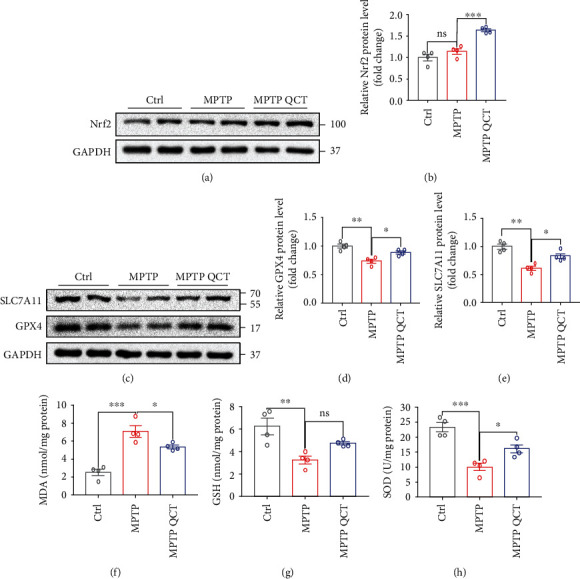
QCT inhibited ferroptosis in PD mouse models by elevating Nrf2 expressions. (a) Representative immunoblots for the Nrf2 protein in SN of Ctrl, MPTP, and MPTP QCT groups. (b) Quantification of Nrf2 proteins in SN (*n* = 4). (c) Representative immunoblots for SLC7A11 and GPX4 proteins in SN of Ctrl, MPTP, and MPTP QCT groups. (d, e) Quantification of SLC7A11 and GPX4 proteins in SN (*n* = 4). (f, g) MDA, GSH, and SOD levels in SN tissues of Ctrl, MPTP, and MPTP QCT groups (*n* = 4). Data are presented as mean ± SEM. One-way ANOVA followed by Tukey's post hoc test. ^∗^*p* < 0.05, ^∗∗^*p* < 0.01, and ^∗∗∗^*p* < 0.001; ns: no significant difference.

To investigate the underlying mechanisms by which QCT protects dopaminergic neurons in SNc, Nrf2 protein levels were assessed. There were no significant differences in Nrf2 protein levels between saline and MPTP treatment groups. However, after QCT treatment, Nrf2 protein levels were markedly elevated, compared to MPTP treatment alone (Figures 7(a) and 7(b)). In addition, GPX4 and SLC7A11 protein levels were downregulated by MPTP treatment, while QCT significantly suppressed these changes, consistent with *in vitro* results (Figures 7(c)–7(e)). Since lipid peroxidation is an essential characteristic of ferroptosis, MDA, GSH, and SOD levels in the SN of mice were measured. Following MPTP treatment, QCT treatment markedly inhibited MDA levels in SN, indicating that QCT inhibits the lipid peroxidation process (Figure 7(f)). Additionally, GSH and SOD levels were decreased following MPTP treatment and these effects were reversed by QCT treatment (Figures 7(g) and 7(h)). Collectively, these results showed that QCT inhibited lipid peroxidation by activating the Nrf2 protein.

## 4. Discussion

**Figure 8:**
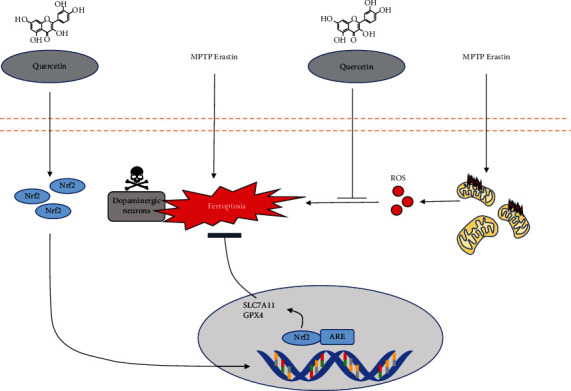
Schematic hypothesis of the antiferroptosis effect of QCT in Parkinson's disease. MPP^+^- and MPTP-induced ferroptosis is involved in midbrain dopaminergic neuronal death. QCT positively regulated SLC7A11 and GPX4 protein levels by activating the Nrf2 pathway. Furthermore, QCT alleviates mitochondrial dysfunction and decreases mitochondrial ROS generation, which is a core factor for ferroptosis.

We found that QCT protected against MPP^+^/MPTP-induced death of dopaminergic neurons in PD *in vitro* and *in vivo*. QCT decreased cell death following treatment with specific ferroptosis inducers (Erastin and RSL-3) or MPP^+^ by inhibiting ferroptosis and improving mitochondrial functions. Moreover, QCT ameliorated behavioral impairments and the loss of dopaminergic neurons in MPTP models. At the molecular level, QCT inhibited ferroptosis by activating the Nrf2 protein, which transcriptionally regulates the expression of downstream ferroptosis-associated proteins, such as GPX4 and SLC7A11 (Figure 8). In summary, our results demonstrate a previously unrecognized mechanism by which QCT inhibits MPP^+^-/MPTP-induced ferroptosis in PD, implying that QCT is a novel protective agent that can delay PD progression.

Various potential mechanisms, such as neuroinflammation, mitochondrial dysfunction, alpha-synuclein misfolding, and aggregation, may be involved in PD onset and progression [34]; however, the exact mechanism is not well understood. Due to the death of dopaminergic neurons in SNc in PD patients, there is a need to establish effective strategies to stop or slow down the progressive death of these neurons. In PD, apoptosis is prevalent; however, other morphological types of cell death have been shown to coexist in brains of PD patients [35]. Ferroptosis has significant implications in several neurologic diseases, including PD, Alzheimer's disease (AD), and ischemic and hemorrhagic stroke [36]. Ferroptosis, which is caused by iron overload in PD, is more initial than apoptosis [37]. Thus, inhibition of ferroptosis may be a promising target for PD treatment.

In our study, we found that QCT significantly inhibited Erastin and RSL-3-induced ferroptosis in M17, PC12, and SH-SY5Y cells and markedly suppressed Erastin-induced ROS generation. These findings indicate that QCT can inhibit ferroptosis. Next, we established that QCT activated Nrf2 protein expression, a transcription factor that is expressed in neurons, microglia, and astrocytes [38–40]. Under normal conditions, Nrf2 is sequestrated by Kelch-like ECH-associated protein 1 (keap1) in the cytoplasm and is quickly degraded by proteosomes to maintain low intracellular levels. However, in response to stress, Nrf2 translocates to the nucleus to transcriptionally activate downstream proteins [38]. Aberrant Nrf2 expression exacerbates Parkinson's pathology and behavioral disorders [41]. Moreover, overexpressed Nrf2 delays PD pathology and alpha-synuclein aggregation [42]. Interestingly, Nrf2 inhibits ferroptosis by transcriptionally regulating downstream ferroptosis-related proteins [24]. Thus, we postulated that QCT inhibits ferroptosis by activating the Nrf2 protein. Consistent with our postulation, our results showed that QCT treatment increased Nrf2 protein levels and its downstream proteins, including GPX4 and SLC7A11 protein levels *in vitro* and *in vivo*. GPX4 is an intracellular antioxidant enzyme that inhibits lipid peroxidation in the cell membrane [6] while SLC7A11 is a light chain subunit of cystine-glutamate antiporter, which can transfer cystine into the cell to synthesize glutathione [43]. To determine whether QCT relies on the Nrf2 pathway to inhibit ferroptosis in PD models, it was realized that Nrf2 protein knockdown compromised the effects of QCT.

In addition, QCT restored the mitochondrial morphology, increased mitochondrial membrane potential and mass, and reduced mitochondrial ROS production. The mitochondria are closely associated with ferroptosis [44, 45]. Moreover, rescuing the mitochondria prevented ferroptosis in different cell types [21, 33, 46]. ROS, which is mainly generated by the mitochondria, is a vital factor in ferroptosis. In this study, we found that QCT protects against MPP^+^-induced ferroptosis by restoring mitochondrial functions and scavenging for mitochondrial ROS.

This study has some limitations. Ferroptosis is often associated with iron metabolism disorders [5]. We found that QCT upregulated FTH protein expressions. The FTH protein, which maintains iron homeostasis by binding ferrous iron, is regulated by the cargo protein nuclear receptor coactivator 4 (NCOA4) that binds FTH and transfers it into the lysosomes for degradation. This process is called ferritinophagy [47–49]. Therefore, QCT inhibits ferritinophagy, which should be evaluated further. The effects of QCT on iron metabolism should also be investigated further. The loss of dopaminergic neurons may be caused by various forms of cell death. Therefore, studies should determine whether QCT affects other forms of cell death, apart from ferroptosis and apoptosis.

In conclusion, QCT prevented the death of dopaminergic neurons by inhibiting ferroptosis. Mechanistically, QCT inhibited ferroptosis of dopaminergic neurons by activating the Nrf2 protein. Further experimental and clinical research should be performed to evaluate the effectiveness and safety of QCT in PD.

## Data Availability

The data set generated in this study is available from the corresponding authors upon request.

## Supplementary Materials

Supplementary Materials Figure S1: the viabilities of M17, PC12, and SH-SY5Y cells after treatment with different QCT concentrations.
