# Supplementary figures and images for: Quercetin Protects against MPP+/MPTP-Induced Dopaminergic Neuron Death in Parkinson's Disease by Inhibiting Ferroptosis
Source: Oxid Med Cell Longev. 2022 Sep 5;2022:7769355. doi: 10.1155/2022/7769355 (PMC9467739; doi:10.1155/2022/7769355)

## Slide 1
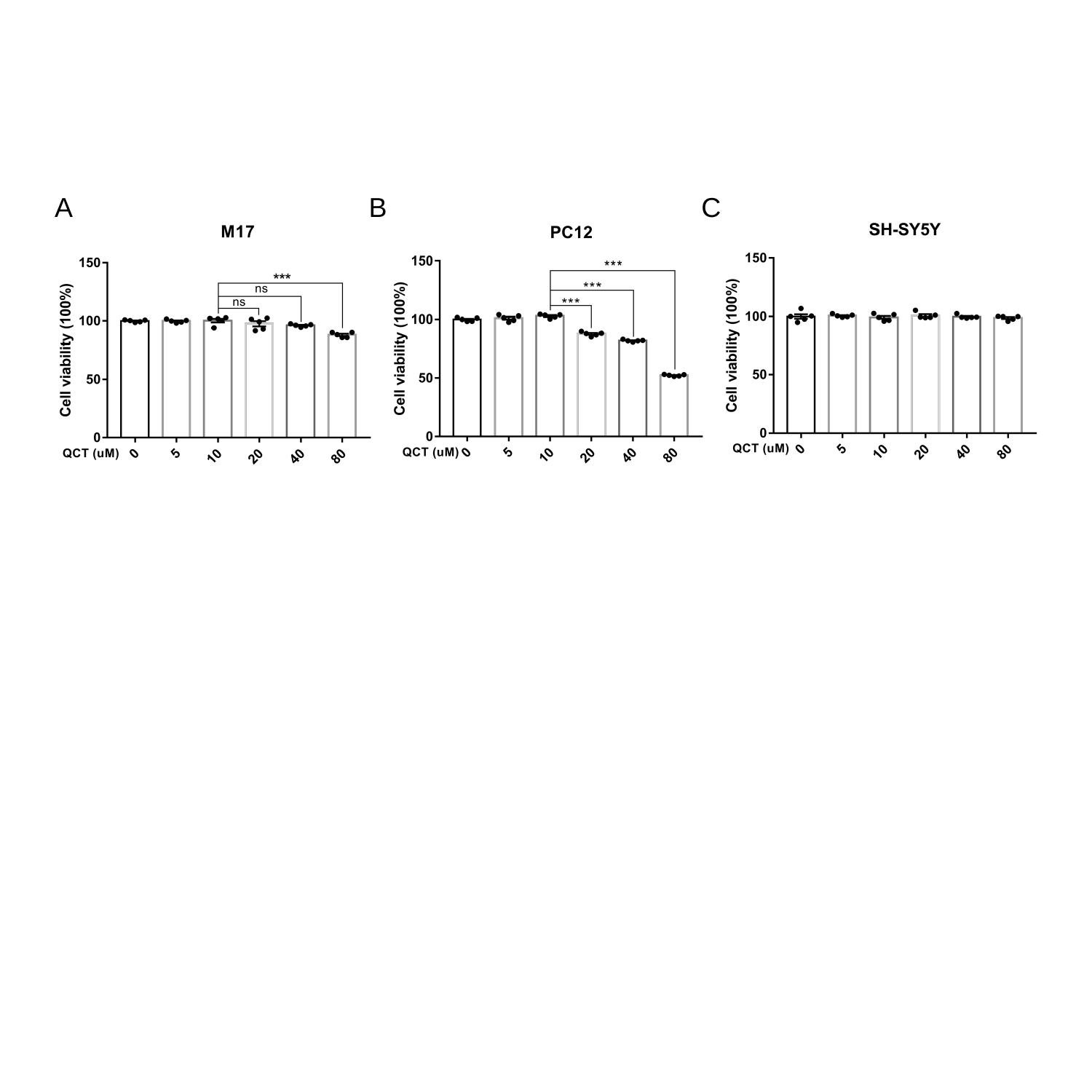

A
B
C

Supplement: Supplementary Materials — Figure S1: the viabilities of M17, PC12, and SH-SY5Y cells after treatment with different QCT concentrations. [file 7769355.f1.pptx]
